# Pro-inflammatory immune responses are associated with clinical signs and symptoms of human anaplasmosis

**DOI:** 10.1371/journal.pone.0179655

**Published:** 2017-06-19

**Authors:** Anna M. Schotthoefer, Steven J. Schrodi, Jennifer K. Meece, Thomas R. Fritsche, Sanjay K. Shukla

**Affiliations:** 1 Marshfield Clinic Research Institute, Marshfield Clinic, Marshfield, Wisconsin, United States of America; 2 Marshfield Labs, Marshfield Clinic, Marshfield, Wisconsin, United States of America; 3 Microbiology Department, University of Wisconsin-La Crosse, La Crosse, Wisconsin, United States of America; Kansas State University, UNITED STATES

## Abstract

Human anaplasmosis (HA) is an emerging tick-borne disease that may present as a mild flu-like illness or a life threatening, sepsis-like condition. Although disease severity is hypothesized to relate to immunopathology and immune dysfunction in humans, studies to directly measure immune responses in infected humans have been very limited. We quantified cytokines in 80 confirmed HA patients using a multiplex chemiluminescence immunoassay system and compared similarly measured responses in 1000 control subjects. Pro-inflammatory cytokines were significantly elevated in HA patients (all seven p<0.0001). Interferon gamma (IFN-γ) concentrations were particularly high, with average concentrations 7.8 times higher in the HA patients than the controls. A subset of cytokines consisting of IL-1β, IL-8, IL-6, TNF-α, and IL-10 was also coordinately high and significantly associated with severity of thrombocytopenia in HA patients. Patients with infections in the very acute stage (≤ 4 days ill) tended to have the highest IFN-γ, IL-12p70, and IL-2 levels. Higher concentrations of IL-13 and IL-5 were associated with diarrhea and vomiting. Our findings support a pathophysiological role for a pro-inflammatory response in HA, especially with regard to the modulation of hematopoiesis and subsequent hematopoietic complications.

## Introduction

Human anaplasmosis (HA) is caused by the emerging rickettsial, tick-borne pathogen, *Anaplasma phagocytophilum* in the USA, Europe, and Asia [1, 2]. The bacterium is an obligate intracellular pathogen, which proliferates primarily in vacuoles of neutrophils in humans and other mammalian hosts [3, 4]. Clinical presentation of the disease varies in humans from an asymptomatic or mild flu-like illness to a severe septic-like syndrome, with potential complications ranging from acute respiratory distress, rhabdomyolysis, and death [1, 5–7]. Common clinical signs and symptoms of HA include fever, chills, headache, myalgia, thrombocytopenia, leukopenia, and elevated hepatic transaminase levels [2, 6]. Approximately 36% of cases in the USA are reported to experience life-threatening complications [8].

The basis for severe disease and the mechanisms that account for the wide spectrum of clinical severity observed in human patients remain poorly understood [9]. Evidence from infections in animal models suggests severity of disease is mediated by the immune response, and that pathogenesis is related to an uncontrolled, innate inflammatory response rather than bacterial load [9–11]. Production of the pro-inflammatory cytokine, interferon gamma (IFN-γ), in particular, appears to play a major role in the immunopathology of the disease in animals [12, 13]. The hypothesis that severity of disease in humans also is largely a function of immune-dysfunction is supported by histologic observations of focal necroses and inflammatory cell aggregates in the absence of significant numbers of the bacterium in the liver and lungs of severely ill HA patients [3]. Elevated levels of IFN-γ, IL-12p70, and the anti-inflammatory cytokine IL-10 have also been detected in HA patients [14, 15].

In this study, we aimed to expand upon the few previous investigations that measured cytokines in HA patients to 1) further delineate the cytokine response in humans and determine if it is predominately characterized by pro-inflammatory, Th1 cytokines as previously suggested and 2) assess cytokine responses in relation to patient characteristics and the clinical manifestations of the disease. Understanding how severity of disease is related to the immune response will be critical for adopting therapies that act to suppress specific immune responses to the treatment of severe HA.

## Materials and methods

### Ethics statement

Waivers of informed consent and authorization to use patient samples and data and all research activities related to this study were reviewed and approved by the Marshfield Clinic Research Institute Institutional Review Board.

### Patient specimen and data collection

The HA specimens (n = 80) used in this study were leftover clinical specimens from patients tested for HA at the Marshfield Clinic and stored at -80°C. All HA infections in patients were confirmed using the real-time PCR assay described in [16, 17], and as performed for clinical diagnosis by Marshfield Labs. Briefly, the assay is designed to amplify and detect a segment of the heat shock protein operon *groEL* in the three closely related organisms: *A*. *phagocytophilum*, *Ehrlichia chaffeensis*, and *Ehrlichia ewingii*; it also amplifies the target in *Ehrlichia* sp. Wisconsin [18]. Differentiation of the related species is done using melting curve analysis [17, 18].

The control samples used in our study were from a cohort of subjects enrolled in the eMERGE (electronic Medical Records and Genomics) Network’s Marshfield Clinic site [19]. This cohort consists of patients who are primarily of Caucasian decent and ≥ 50 years old that have been genotyped on Illumina’s Human 660W-Quad Array. The cohort includes a subpopulation of 1000 patients that had their cytokine profiles separately analyzed using the same multiplex immunoassay system that was used on the HA patients. Inclusion criteria for the subpopulation were 1) no evidence of elevated temperature at the time of sample collection, 2) no evidence of elevated white blood cell (WBC) count at the time of sample collection, 3) no immunotherapy at the time of sample collection, and 4) that the individual had no evidence of a divergent genetic background using the 660W-Quad array genome data. Although not specifically tested for HA, participants in this cohort were very unlikely to be infected with *A*. *phagocytophilum* at the time of their sample collections because of the criteria to exclude those with an acute inflammatory illness.

Data for the HA patients were abstracted from the medical record and included age, gender, number of days between symptom onset and clinical sample collection, signs and symptoms reported by the patient or detected during physical exams, and the results of laboratory tests, including blood cell counts, liver enzyme assays, urine analyses, and other infectious disease diagnostic tests, as collected for medical care associated with the illness. We also determined whether patients were hospitalized, and if so, the length of the hospital stay, as well as if the patient experienced any other severe complications, such as admission to an intensive care unit, or death. None of the patients included in the study had initiated antibiotic treatment prior to serum collection or had positive laboratory tests for Lyme disease or babesiosis, or presented with a rash consistent with erythema migrans, which would indicate possible co-infection with Lyme disease.

### Quantification of cytokine profiles

Cytokines were quantified in duplicate serum samples and averaged for the HA patients and in single samples for the controls using a multiplex immunoassay system according to manufacturer recommendations (Meso Scale Diagnostics, Gaithersburg, MD). The human Th1/Th2 10-Plex assay kit, which measures concentrations of IFN-γ, IL-1β, IL-2, IL-4, IL-5, IL-8, IL-10, IL-12p70, IL-13, and TNF-α, was used on 41 of 80 HA patients. In addition, we measured the concentrations of IL-6 in a single-plex IL-6 assay in these samples. The remaining 39 HA patients in the study and the 1000 controls had their cytokines analyzed using the 7-Plex pro-inflammatory panel, which measured IFN-γ, IL-1β, IL-6, IL-8, IL-10, IL-12p70 and TNF-α. Calibrator samples included in the assay kits were used to generate standard curves on which the cytokine concentrations in our samples were calculated.

### Statistical analyses

We used log-transformed cytokine concentrations in our analyses. We compared the concentrations of each cytokine that was measured in our HA patients and controls (e.g., IFN-γ, IL-1β, IL-6, IL-8, IL-10, IL-12p70 and TNF-α) using t-tests and adjusted the p-values for the multiple tests by bootstrap resampling of 10,000 samples [20]. We also visually examined the overall cytokine responses in the HA patients compared to the controls using Principal Components Analysis (PCA).

We used t-tests with bootstrap resampling (10,000 samples) to evaluate possible univariate associations between cytokine concentrations and patient categorical characteristics and the presence or absence of various clinical signs and symptoms within our HA patients (e.g., see S1 Table). PCA was then used to examine the multivariate correlations among the cytokines and to collapse the dimensionality of the HA cytokine data. We took the principal component scores from these analyses and conducted stepwise regression analyses to further explore associations among cytokine responses and patient characteristics and clinical signs and symptoms. Finally, we assigned cytokine concentrations to ordinal categories based on the quartiles calculated from the HA patient data (e.g., concentrations < 25% = rank 1; concentrations ≥ 25% to < 50% = rank 2; concentrations ≥ 50% to < 75% = rank 3; concentrations ≥ 75% = rank 4) and generated heatmaps to visualize variation in the cytokine responses. We counted the number of cytokines ranked as 4 for each patient, and then compared these counts using Wilcoxon-Mann-Whitney or Kruskal-Wallis tests to examine categorical patient and clinical variables in relation to an overall, high cytokine response [21]. We used the Division of Allergy and Infectious Diseases Adverse Events Grading Table [22] for defining categories of severity of thrombocytopenia and leukopenia to examine in relation to HA cytokine responses in these analyses. All analyses were conducted in SAS 9.4. Significance was defined as p-values ≤ 0.05, except in the stepwise regression analyses in which the entry and stay significance levels were defined at 0.15.

## Results

Average age of our HA cohort was 54, with ages ranging between 2 and >89 (Table 1). Our sample included 6 pediatric patients (ages < 18). Fifty (62.5%) of the HA patients were male. Our HA patients were primarily in the acute phase of infection, with 53 (66.3%) of the patients ill for 4 or fewer days (Table 1). Symptoms presented by the patients were consistent with other reports for HA, with the majority of patients presenting with a febrile illness. There also were high percentages of patients with clinical evidence of leukopenia, thrombocytopenia, and elevated liver enzymes, and 100% of 25 patients evaluated had elevated concentrations of serum C-reactive protein at the time of presentation (Table 1).

**Table 1:** Demographics and clinical characteristics of patients with human anaplasmosis.

	HA patients		Controls	
	Sample size	N (%)	Sample size	N (%)
Age (Mean± 1 SD)	80	54.2±20.1	1000	64.3±11.3
Gender (male)	80	50 (62.5)	1000	439 (43.9)
Laboratory diagnostics ^a^				
Positive serology	44	17 (38.6)		
Positive blood smear	25	14 (56.0)		
Duration of illness	80			
≤ 4 days		53 (66.3)		
5–14 days		21 (26.3)		
15–32 days		6 (7.5)		
Clinical course				
Hospitalized	80	19 (23.8)		
Positive for symptoms:	80			
Fever/sweats/chills		76 (95.0)		
Fatigue		51 (63.8)		
Headache		48 (60.0)		
Myalgias		48 (60.0)		
Cough		19 (23.8)		
Nausea		23 (28.8)		
Vomiting		12 (15.0)		
Diarrhea		12 (15.0)		
Decreased appetite		28 (35.0)		
Leukopenia ^b^	74	43 (58.1)		
Severity of leukopenia ^c^	72			
Normal-not severe		60 (81.1)		
Grade 1		5 (6.8)		
Grade 2		7 (9.5)		
Grade 3		0		
Grade 4		0		
Thrombocytopenia ^d^	74	52 (70.3)		
Severity of thrombocytopenia ^e^				
Normal-not severe		25 (33.8)		
Grade 1		16 (21.6)		
Grade 2		23 (31.1)		
Grade 3		9 (12.2)		
Grade 4		1 (1.4)		
Anemia ^f^	74	14 (18.9)		
Elevated AST liver enzymes ^g^	51	30 (58.8)		
Elevated ALT liver enzymes ^h^	54	16 (29.6)		
Elevated C-reactive protein ^i^	25	25 (100)		
Hematuria or hemoglobinuria	49	32 (65.3)		
Evidence of urinary tract infection (= microbial growth on urine culture)	29	12 (41.4)		

^**a**^ All 80 patients had a positive PCR test for *Anaplasma phagocytophilum*.

^b^ Defined as WBC count < 4.1 x 10^9^ cells/L for adults, or < 4.5 x 10^9^ cells/L for children

^c^ Based on DAIDS AE Grading Table [22]; Normal-not severe WBC count < 4.1–2.5 x 10^9^; Grade 1 (mild) WBC count < 2.5–2.0 x 10^9^ cells/L; Grade 2 (moderate) WBC count < 2.0–1.5 x 10^9^ cells/L; Grade 3 (severe) WBC count <1.5–1.0 x 10^9^/L; Grade 4 (potentially life-threatening) WBC count <1.0 x 10^9^/L. Two patients had elevated WBC counts (> 11 x 10^9^ cells/L) and were excluded from analyses pertaining to leukopenia.

^d^ Defined as platelet count < 175 x 10^9^ cells/L for adults, or < 150 x 10^9^ cells/L for children.

^e^ Based on DAIDS AE Grading Table [22]; Normal platelet count ≥ 125 x 10^9^/L; Grade 1 (mild) <125–100 x 10^9^/L; Grade 2 (moderate) platelet count <100–50 x 10^9^/L; Grade 3 (severe) platelet count <50–25 x 10^9^/L; Grade 4 (potentially life threatening) <25 x 10^9^/L. Three patients had platelet counts that met the definition of thrombocytopenia as defined in d, but which were considered normal-not severe based on DAIDS criteria.

^f^ Defined as hemoglobin < 11.5 g/dL for children < 10 years, < 12.5 g/dL for males 10–17 years, < 12.0 g/dL for females 10–17 years, < 12.9 for adult males, or < 11.7 g/dL for adult females.

^g^ Reference ranges varied by patient age and instrumentation used in assay; in our study, high values ranged between 42–266 U/L and low values ranged between 16–46 U/L.

^h^ Reference ranges varied by patient age and instrumentation used in assay; in our study, high values ranged between 79–269 U/L and low values ranged between 11–73 U/L.

^i^ Defined as C-reactive protein concentrations > 1.0 mg/dL.

**Fig 1:**
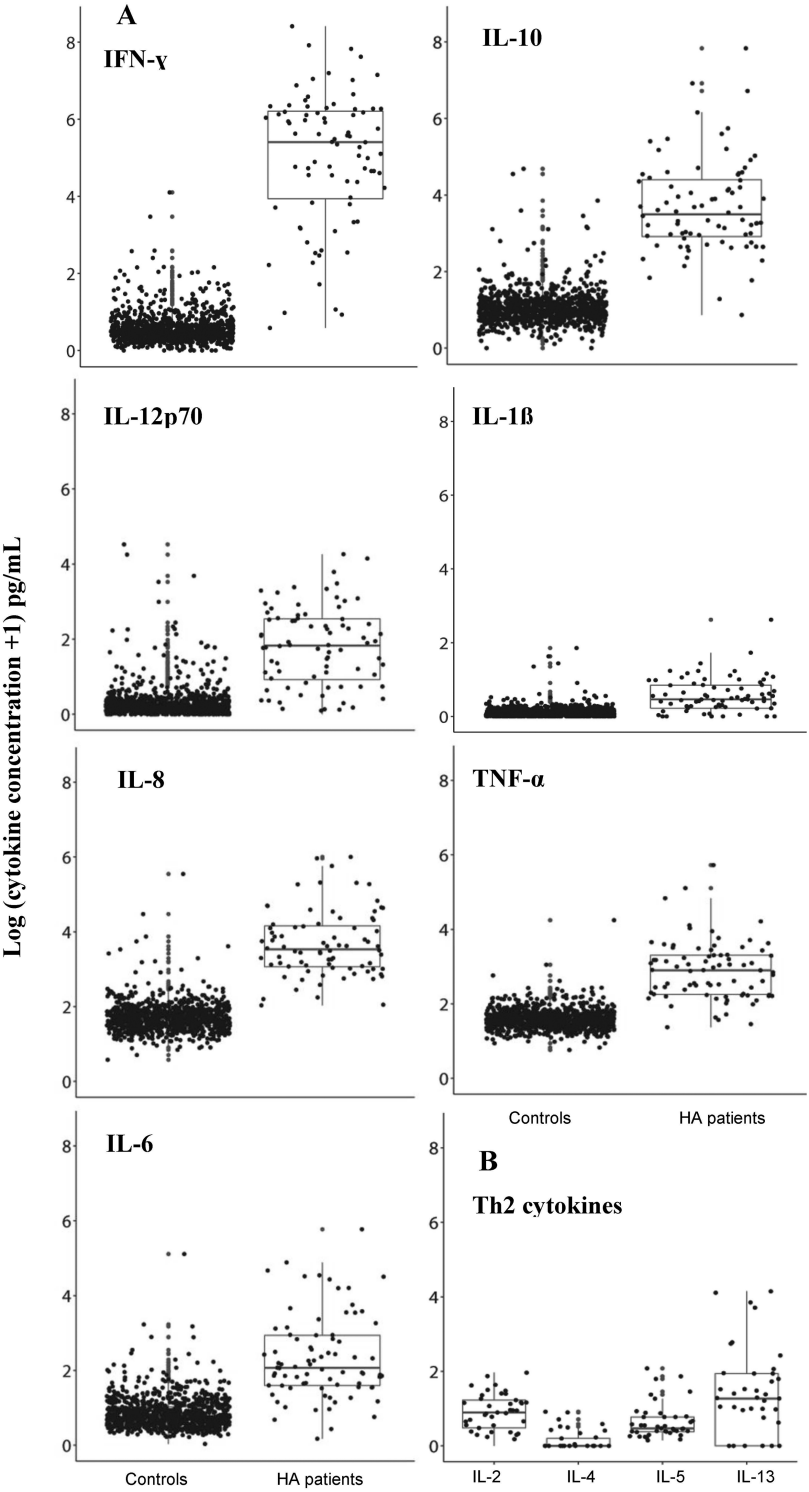
Comparisons of cytokines between HA patients and controls. Logarithmic concentrations (pg/mL) of cytokines measured in the HA patients (n = 80) compared to controls (n = 1000) for the Th1/pro-inflammatory cytokines (A) and the Th2 cytokines measured in 41 of the HA patients (B). Boxplots representing the 25%, 50%, and 75% quartiles and the 95% confidence intervals of the medians for each sample group are displayed.

All seven of the Th1/pro-inflammatory cytokines were significantly higher in our HA patients compared to the controls (Table 2; Fig 1). IFN-γ was particularly elevated, with the average concentration 7.8 times higher in the HA patients compared to the controls. Average concentrations of IL-10, IL-12p70, and IL-1β also were more than 2 times higher in the HA patients than the controls. Though we did not have comparable control data for IL-2, IL-4, IL-5, or IL-13, their concentrations tended to be lower than the Th1/pro-inflammatory cytokines measured in the HA patients and all except IL-4 were detected at concentrations significantly greater than the lower limits of detection for the assays in the HA patients (Table 2).

**Table 2:** Averages (1 S.D.) and comparisons of the log-transformed cytokine concentrations (pg/mL) detected in the HA patients and controls included in the study.

Cytokine	HA Patients (n = 80)	Controls (n = 1000)	p-value^a^
IFN-γ	5.00 (1.746)	0.571 (0.386)	**< 0.0001**
IL-10	3.70 (1.237)	1.055 (0.423)	**< 0.0001**
IL-12p70	1.79 (1.089)	0.297 (0.409)	**< 0.0001**
IL-1β	0.55 (0.467)	0.101 (0.141)	**< 0.0001**
IL-8	3.67 (0.878)	1.707 (0.378)	**< 0.0001**
TNF-α	2.89 (0.798)	1.585 (0.262)	**< 0.0001**
IL-6	2.34 (1.124)	0.849 (0.410)	**< 0.0001**
	(n = 41)		
IL-2	0.90 (0.505)		**<0.0001**
IL-4	0.14 (0.263)		0.052
IL-5	0.66 (0.471)		**<0.0001**
IL-13	1.34 (1.184)		**0.005**

^a^ Comparisons for the cytokines measured in both the HA patients and controls were made using t-tests with bootstrap resampling to adjust p-values for multiple tests. For the cytokines measured only in the HA patients, comparisons were between the means and the lower limits of detections provided by the manufacturer of the assay kits. P-values ≤ 0.5 are shown in bold.

Within the HA patients, we found no significant differences in the cytokine concentrations between males and females or within older age groups (S1 Table). Patients in the earliest stage of infection (ill ≤ 4 days) had significantly higher IFN-γ, IL-12p70, and IL-2 concentrations (all adjusted p ≤ 0.048). Patients with morulae detected on blood smears had higher IL-10, IL-1β, and IL-6 concentrations (all adjusted p ≤ 0.030). Hospitalized patients had higher IL-10 and TNF-α concentrations (both adjusted p ≤ 0.036) (S1 Table). Few differences in cytokine concentrations were detected in association with specific symptoms, though vomiting and diarrhea were associated with higher IL-13 and IL-5 (both adjusted p ≤ 0.023) and thrombocytopenia was associated with higher IFN-γ, IL-10, IL-1β, IL-8, and IL-6 (all adjusted p ≤ 0.031) (S1 Table).

**Fig 2:**
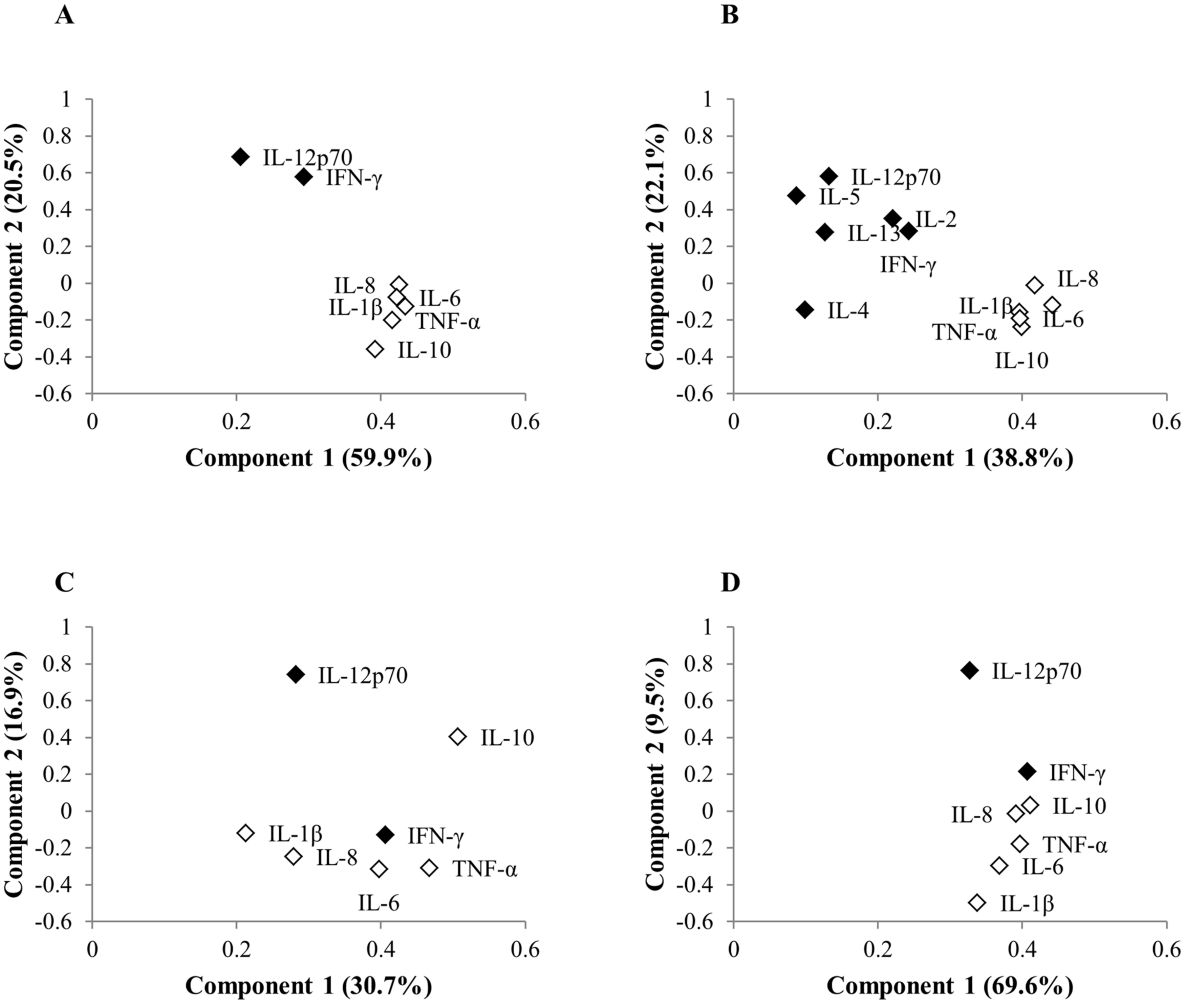
Ordination plots for cytokine PCAs. Ordination of the Th1/pro-inflammatory cytokines measured in 80 HA patients (A), the Th1/pro-inflammatory and Th2 cytokines measured in 41 HA patients (B), the Th1/pro-inflammatory cytokines measured in controls (C), and the Th1/pro-inflammatory cytokines measured in all HA patients and controls (D). Plots for component 1 versus 2 scores only are displayed. Open symbols represent the cluster of highly correlated, pro-inflammatory cytokines observed in the HA patients.

**Fig 3:**
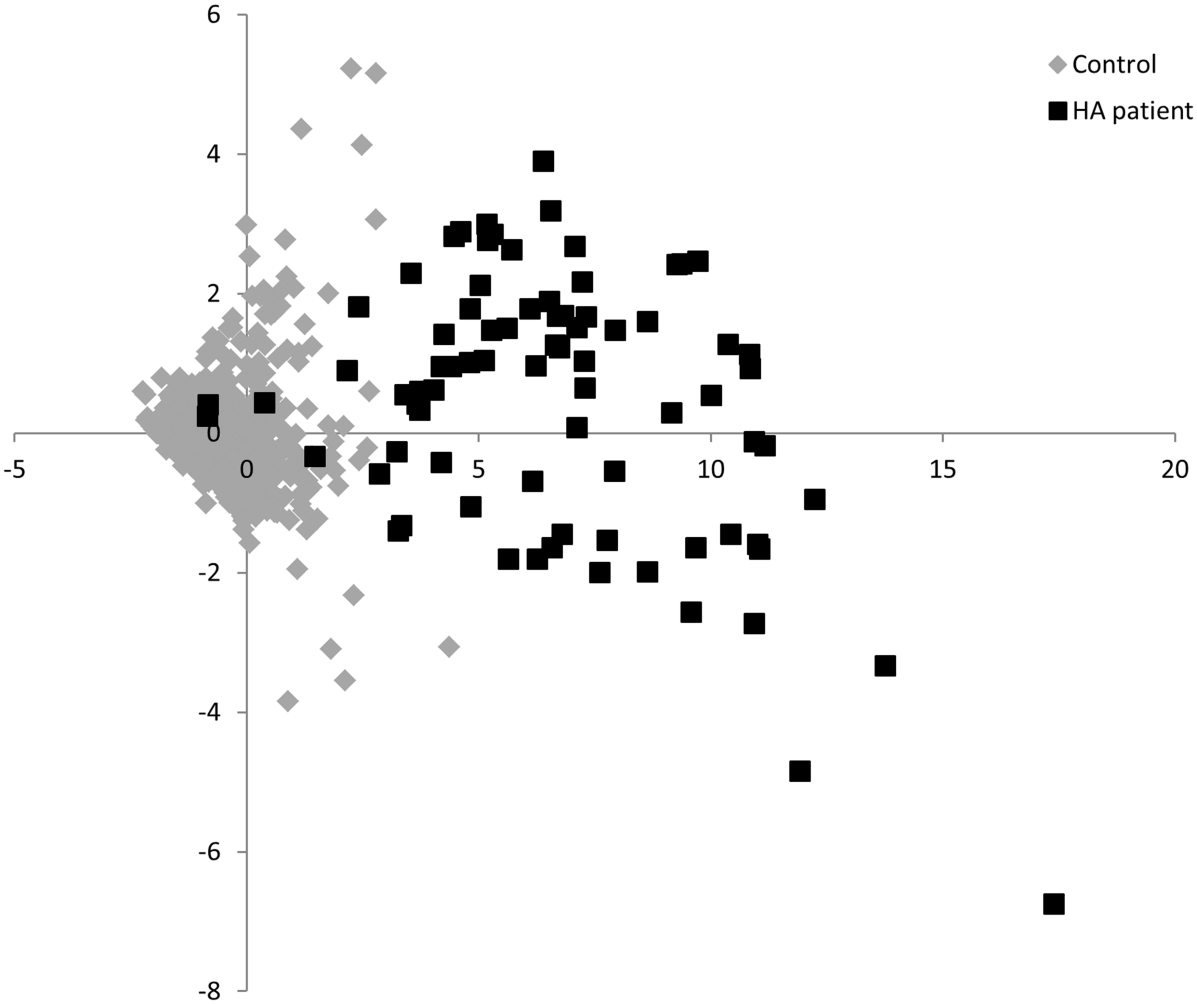
Ordination of patients. Ordination of HA patients (n = 80) and controls (n = 1000) based on the component 1 and component 2 scores obtained from the PCA done on the seven Th1/pro-inflammatory cytokines measured in the study.

We observed significant, positive correlations among the pro-inflammatory cytokines, IL-8, IL-6, TNF-α, and IL-1β within our HA patients (all Spearman r ≥ 0.60, p < 0.0001) as well as in the controls (all Spearmen r ≥ 0.11, p < 0.0003) (S2 Table), and this subset of cytokines explained the largest amount of variation in the cytokine profiles in both groups, defining the first component scores in our PCA analyses (eigenvectors = 0.39–0.43 and 0.21–0.47 for HA patients and controls, respectively; Fig 2, S3–S6 Tables). We observed differences in the associations among IL-10, IFN-γ, and IL-12p70 in the HA patients and controls. IL-10 was positively correlated with IFN-γ and IL-12p70 in the controls (Spearman r = 0.23 and 0.28, respectively, both p < 0.0001), whereas it was positively correlated with the subset of pro-inflammatory cytokines in the HA patients (Spearman r = 0.60–0.73, all p < 0.0001), and therefore was also positively correlated with the component 1 scores for the PCAs done on the HA patient data (eigenvectors = 0.39 and 0.40; Fig 2; S2–S4 Tables). The cytokine, IL-12p70, separated from the pro-inflammatory subset and explained much of the variation in the second component scores (eigenvectors = 0.58–0.69 and 0.74 for HA patients and controls, respectively; Fig 2, S3–S5 Tables). In the HA patients, IFN-γ was more strongly associated with IL-12p70, and therefore component score 2, than the subset of pro-inflammatory cytokines (Fig 2; eigenvectors = 0.58 versus 0.29 and 0.29 versus 0.24, S3 and S4 Tables, respectively). When we ordinated our HA patients and controls, we saw a clear separation of the two groups (Fig 3).

In the PCA done on data from the HA patients that also had the Th2 cytokines measured (n = 41), we saw IL-5, IL-2, and IL-13 grouping with IL-12p70 and IFN-γ (Fig 2, S4 Table). The scree plot for this analysis suggested that the first four components should be retained (86.9% of total variance explained). The third component (17.5% variance) was most positively associated with IL-13 and IL-5 (eigenvectors = 0.58 and 0.41, respectively) and negatively associated with IFN-γ and IL-2 (eigenvectors = -0.39 and -0.37, respectively). The fourth component (8.6% variance) was associated with IL-4 and IFN-γ (eigenvectors = 0.85 and 0.31, respectively; S4 Table).

**Fig 4:**
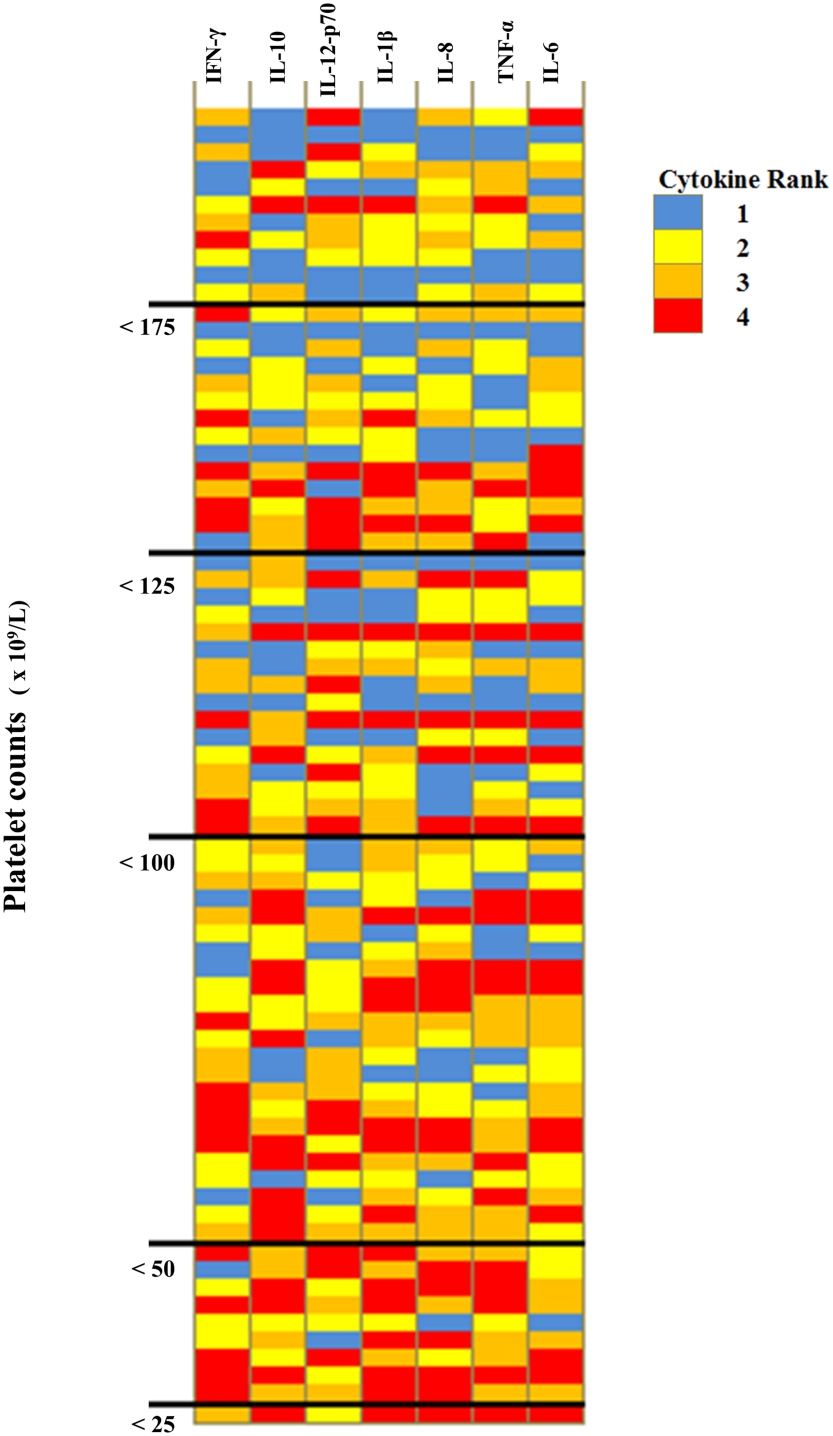
Severity of thrombocytopenia and cytokine responses. Heatmap of the Th1/pro-inflammatory cytokines measured in HA patients arranged in order of platelet counts. Colors represent the categorical ranks assigned to cytokine concentrations based on quartiles from the HA patients: blue (1) < 25^th^ percentiles; yellow (2) between the 25^th^ and 50^th^ percentiles; orange (3) between the 50^th^ and 75^th^ percentiles; and red (4) ≥ 75^th^ percentiles.

Stepwise regression analyses utilizing the component scores from the PCA analyses conducted on the HA patients as dependent variables corroborated the results from our univariate analyses. Platelet count was the only variable significantly associated with component 1 scores derived from the PCA done on HA patients with Th1 and Th2 cytokine data (p = 0.020) as well as the PCA done on the patients with only the Th1/pro-inflammatory cytokine data (p = 0.005). Being ill for ≤ 4 days was associated with component 2 scores (p = 0.035 for Th1/Th2 cytokine data and p = 0.0007 for Th1/pro-inflammatory data). For the HA PCA that included Th1/Th2 cytokines, diarrhea (p = 0.006) and vomiting (p = 0.078) were associated with component 3 scores, but none of our clinical variables were found to be significantly related to the component 4 scores. Thrombocytopenia tended to be more severe in patients with a higher number of highly elevated (e.g., rank 4) Th-1/pro-inflammatory cytokines (Kruskal-Wallis test, p = 0.041; Fig 4). None of our models suggested that gender, age, or hospitalization were significantly related to the cytokine levels measured in HA patients.

## Discussion

Our results support the hypothesis that *A*. *phagocytophilum* infections stimulate a Th1/pro-inflammatory cytokine response in humans. As reported in other studies [14, 15], IFN-γ was particularly elevated in the HA patients, with 74% of our patients having IFN-γ concentrations that were higher than the maximum detected in our controls. Concentrations of IFN-γ, although positively correlated with other Th1 promoting cytokines in our study, were more closely associated with concentrations of IL-12p70 and IL-2 in HA patients, and these three cytokines (IFN-γ, IL-12p70, and IL-2) were significantly higher in patients in the very acute stage of infections (S1 and S2 Tables), consistent with their known importance in early inflammatory responses. They play key roles in activating macrophages, directing Th cell differentiation, and in activating the adaptive immune response in bacterial, viral, and parasitic infections [23, 24]. Indeed, IFN-γ has been demonstrated to play an important role in the control of *A*. *phagocytophilum* infections in mice and horses [12, 13].

We also report for the first time, evidence that TNF-α and IL-1β are elevated in HA. Previous studies that had measured cytokines in HA patients failed to detect elevation in these cytokines, probably because of the different immunoassay methods used [14, 15]. Importantly, TNF-α and IL-1β were part of a subset of cytokines that also included IL-6, IL-8, and IL-10, which were significantly more elevated in HA patients and were strongly associated with each other. These cytokines are associated with febrile illnesses, and evidence of their concomitant elevation is consistent with responses observed in other intracellular pathogens, including *Toxoplasma gondii* [25], *Plasmodium falciparum* [26], and *Mycobacterium tuberculosis* [27]. IL-10 is an anti-inflammatory cytokine that is produced by many cell types including macrophages, dendritic cells (DCs), and various CD4+ and CD8+ T cells [28] and its association with the pro-inflammatory subset in the HA patients, but not in our controls, highlights its important role in regulating the Th1/pro-inflammatory response in HA [9, 13, 28, 29].

The cytokines, IL-13 and IL-5, also were elevated in some HA patients, particularly, those reporting vomiting and diarrhea. These two cytokines are known to be involved in inducing physiological changes in gut epithelial cells. They are involved in the process of expelling helminths from the gut, and are associated with eosinophilic gastrointestinal disorders that result in atopy and food allergies [30]. Thus, the symptoms of vomiting and diarrhea are consistent with their activation. Although they traditionally were thought to be coordinately regulated in Th2 driven responses, a de-coupling of their production and their production by Th1 cells, particularly in association with IFN-γ, has been demonstrated [31]. It also may be possible that IL-13 and IL-5 play a role in the coordination of the adaptive CD4+T cell response that is critical for control of HA infections [32].

Although we were unable to examine immunopathology directly in our HA patients, our evaluation of cytokine responses in relation to clinical presentation of HA revealed a strong association between thrombocytopenia and the concentrations of pro-inflammatory cytokines. Thrombocytopenia, which was present in 70% of our HA patients, is a characteristic sign of *A*. *phagocytophilum* infections in humans suspected of a tick-borne disease illness, as well as in other animals [2, 33, 34]. Leukopenia is also common in HA patients, although we did not detect any differences in cytokine concentrations between patients that were and were not leukopenic, possibly because WBC counts were only mildly suppressed in the majority of our HA patients (Table 1). The mechanisms that underlie hematologic complications in HA infections remain largely unexplored, although they are outcomes commonly associated with infectious diseases [35–37]. Mechanisms of thrombocytopenia include increased platelet sequestration, destruction, or inhibition of megakaryopoiesis [38]. In HA, it is unlikely that the declines in cells are due to antibody-mediated destruction or sequestration of cells, as the declines are observed prior to the development of an acquired immune response [34], rather it is more likely that the decline in platelet numbers is related to the complex interactions that occur between inflammation and hemostasis. Pro-inflammatory cytokines are associated with activation of the coagulation system and platelets are currently being recognized as having an important role in immune responses [39, 40]. Moreover, in vitro studies and studies in mice have demonstrated that *A*. *phagocytophilum* infection results in myelosuppression of bone marrow progenitor cells, as many pro-inflammatory chemokines and cytokines are myelosuppressive [11]. *A*. *phagocytophilum* also appears to directly stimulate the production of chemokines by infected cells that are strong inhibitors of hematopoiesis stem cell proliferation [41].

It has been hypothesized that severe disease in HA may result from excessive IFN-γ and uncontrolled macrophage activation, similar to that observed in reactive hemophagocytic lymphohistiocytosis (rHLH) or macrophage activation syndromes (MAS) [9, 15]. In these syndromes, the cytotoxic effects of activated CD8+ T lymphocytes, NK cells, or NKT cells that normally kill infected cells and ultimately regulate activated macrophages are dysfunctional, leading to cell and tissue injury. The elevated concentrations of IFN-γ, IL-12, IL-1β, and IL-10 that we detected in our HA patients clearly indicate that macrophages and the innate immune responses are activated. However, whether severe disease is related to uncontrolled macrophage activation, and if so, what the etiology of that uncontrolled activation might be, remain unclear (see for instance, [42]). In rHLH and MAS, there appears to be an underlying genetic basis with mutations specifically affecting the exocytosis of cytotoxic effector molecules [43]. These mutations have been detected in people that suffered from severe manifestations of infections with other pathogens that also display pathogenesis consistent with rHLH/MAS [44–46], such that there may be a similar underlying genetic basis to some severe infections in HA. Currently, however, there have been no studies that have examined the genetic susceptibilities of HA patients in relation to disease severity, or that have even specifically measured the cytotoxic activities of CD8+ T or NK cells to determine if their activities vary with disease severity. Moreover, epidemiologic evidence suggests disease severity is related to other patient factors such as age, immune status, or comorbidities [1], factors that might influence a patient’s ability to regulate a non-specific, pro-inflammatory immune response.

In vitro studies indicate that *A*. *phagocytophilum* possesses multiple mechanisms for subverting neutrophil antimicrobial defenses and weakening or delaying release of pro-inflammatory cytokines and chemokines from neutrophils, thus the pro-inflammatory cytokines detected in HA patients are likely produced by other cell sources, such as monocytes and NK cells [47]. Studies to identify these sources and to better understand specifically how *A*. *phagocytophilum* infection alters coordination of the innate and adaptive immune responses would lend better insight into the mechanisms of severe HA disease in humans. Furthermore, studies to explore how the genetic variants of *A*. *phagocytophilum* influence disease severity are lacking, despite evidence that there are differences in the ability of different strains to cause disease in humans and animals [48]. In conclusion, although our study provides evidence for a role of a Th1/pro-inflammatory cytokine response in HA, further studies would help determine how these and other cytokines modulate the immunopathology and hematopoietic complications associated with the disease. A better understanding of the mechanisms of pathogenesis may ultimately result in fewer patients requiring hospitalization or that suffer from severe infections by pointing to alternate treatments that might be utilized in the event that antibiotic therapies are not available.

## Supporting information

S1 Table Cytokine responses in relation to HA patient demographic and clinical characteristics. Comparisons in the concentrations of cytokines between HA patients with (Y) and without (N) the characteristics listed. Values are the mean differences (Y–N) and adjusted p-values for each univariate comparison. Significant differences are in bold.
(DOCX)

S2 Table Correlations among circulating cytokines in HA and in control subjects. Correlation matrix for the log-transformed cytokine concentrations measured in HA patients in the upper, right corner of the matrix and for the controls in the lower, left corner of the matrix. Values are Spearman rank correlations. Cells are color-coded according to the p-value.
(DOCX)

S3 Table PCA results for Th1/pro-inflammatory cytokines, HA patients. Summary of the PCA analysis based on concentrations of the Th1/pro-inflammatory cytokines measured in the HA patients (n = 80). Values are the eigenvectors for each cytokine on the retained component scores. Proportions of the variation in the cytokine data explained by the component scores are also indicated.
(DOCX)

S4 Table PCA results for Th1/Th2 cytokines, HA patients. Summary of the PCA analysis based on concentrations of the Th1/Th2 cytokines measured in the HA patients (n = 41). Values are the eigenvectors for each cytokine on the retained component scores. Proportions of the variation in the cytokine data explained by the component scores are also indicated.
(DOCX)

S5 Table PCA results for Th1/pro-inflammatory, controls. Summary of the PCA analysis based on concentrations of the Th1/pro-inflammatory cytokines measured in the controls (n = 1000). Values are the eigenvectors for each cytokine on the retained component scores. Proportions of the variation in the cytokine data explained by the component scores are also indicated.
(DOCX)

S6 Table PCA results for Th1/pro-inflammatory, HA patients and controls. Summary of the PCA analysis based on concentrations of the Th1/pro-inflammatory cytokines measured in the HA patients and the controls (n = 1080). Values are the eigenvectors for each cytokine on the retained component scores. Proportions of the variation in the cytokine data explained by the component scores are also indicated.
(DOCX)
