# Supplementary material for: Pro-inflammatory immune responses are associated with clinical signs and symptoms of human anaplasmosis
Source: PLoS One. 2017 Jun 19;12(6):e0179655. doi: 10.1371/journal.pone.0179655 (PMC5476275; doi:10.1371/journal.pone.0179655)
Supplement: S1 Table — Comparisons in the concentrations of cytokines between HA patients with (Y) and without (N) the characteristics listed. Values are the mean differences (Y–N) and adjusted p-values for each univariate comparison. Significant differences are in bold. (DOCX) [file pone.0179655.s001.docx]

**S1 Table**. **Cytokine responses in relation to HA patient demographic and clinical characteristics.** Comparisons in the concentrations of cytokines between HA patients with (Y) and without (N) the characteristics listed. Values are the mean differences (Y – N) and adjusted p-values for each univariate comparison. Significant differences are in bold.

|  | **IFN-γ** |  | **IL-10** |  | **IL-12p70** |  | **IL-1β** |  | **IL-8** |  | **TNF-α** |  |
| --- | --- | --- | --- | --- | --- | --- | --- | --- | --- | --- | --- | --- |
|  | **Y - N** | ***P*** | **Y - N** | ***P*** | **Y - N** | ***P*** | **Y - N** | ***P*** | **Y - N** | ***P*** | **Y - N** | ***P*** |
| **Age ≥ 50** | -0.038 | 1 | 0.398 | 0.535 | -0.024 | 1 | 0.228 | 0.151 | 0.338 | 0.352 | 0.35 | 0.229 |
| **Age ≥ 60** | -0.191 | 0.994 | 0.403 | 0.516 | 0.139 | 0.983 | 0.214 | 0.179 | 0.257 | 0.617 | 0.297 | 0.381 |
| **Gender (male)** | -0.243 | 0.979 | -0.454 | 0.391 | 0.034 | 1 | -0.115 | 0.771 | -0.095 | 0.995 | -0.186 | 0.81 |
|  |  |  |  |  |  |  |  |  |  |  |  |  |
| **Duration of illness**  **≤ 4 days** | **1.928** | **<0.0001** | -0.204 | 0.963 | **0.784** | **0.0097** | 0.177 | 0.423 | 0.145 | 0.961 | 0.051 | 1 |
|  |  |  |  |  |  |  |  |  |  |  |  |  |
| **Laboratory diagnostics** |  |  |  |  |  |  |  |  |  |  |  |  |
| **Positive serology** | -1.085 | 0.115 | 0.053 | 1 | -0.357 | 0.791 | -0.14 | 0.916 | -0.227 | 0.9 | -0.325 | 0.635 |
| **Positive blood smear** | 0.093 | 1 | **1.773** | **0.0004** | -0.311 | 0.976 | **0.599** | **0.036** | 0.555 | 0.39 | 0.597 | 0.141 |
|  |  |  |  |  |  |  |  |  |  |  |  |  |
|  |  |  |  |  |  |  |  |  |  |  |  |  |
| **Hospitalized** | 0.388 | 0.903 | **0.863** | **0.036** | 0.293 | 0.785 | 0.338 | 0.026 | 0.271 | 0.699 | **0.682** | **0.005** |
| **Clinical manifestations:** |  |  |  |  |  |  |  |  |  |  |  |  |
| **Fever** | 1.402 | 0.076 | 0.416 | 0.819 | 0.687 | 0.221 | 0.214 | 0.569 | 0.359 | 0.673 | 0.617 | 0.096 |
| **Chills** | 0.717 | 0.303 | -0.053 | 0.999 | 0.37 | 0.47 | 0.12 | 0.748 | 0.062 | 1 | -0.032 | 1 |
| **Fatigue** | -0.635 | 0.422 | -0.533 | 0.255 | -0.257 | 0.79 | -0.223 | 0.169 | -0.191 | 0.852 | -0.307 | 0.364 |
| **Headache** | 0.752 | 0.243 | -0.322 | 0.728 | 0.0077 | 1 | -0.0185 | 1 | -0.0641 | 1 | -0.205 | 0.738 |
| **Myalgias** | -0.196 | 0.994 | -0.309 | 0.749 | -0.142 | 0.983 | -0.133 | 0.65 | -0.069 | 0.999 | -0.128 | 0.954 |
| **Cough** | 0.559 | 0.662 | 0.773 | 0.076 | 0.297 | 0.769 | 0.126 | 0.793 | 0.282 | 0.659 | 0.423 | 0.188 |
| **Nausea** | 0.531 | 0.653 | 0.422 | 0.542 | -0.0082 | 1 | 0.233 | 0.18 | 0.303 | 0.533 | 0.488 | 0.062 |
| **Vomiting** | 0.342 | 0.971 | 0.137 | 0.999 | 0.457 | 0.54 | 0.167 | 0.714 | 0.215 | 0.925 | 0.191 | 0.9332 |
| **Diarrhea** | 0.113 | 1 | -0.101 | 1 | 0.376 | 0.721 | 0.056 | 0.999 | -0.071 | 1 | 0.097 | 1 |
| **Decreased appetite** | -0.319 | 0.933 | -0.375 | 0.614 | -0.32 | 0.618 | -0.142 | 0.611 | -0.3 | 0.498 | -0.276 | 0.487 |
| **Leukopenia** | 0.613 | 0.449 | -0.033 | 1 | 0.088 | 0.998 | -0.022 | 1 | -0.025 | 1 | -0.008 | 1 |
| **Thrombocytopenia** | **1.506** | **0.018** | **1.046** | **0.024** | 0.537 | 0.356 | **0.396** | **0.026** | **0.744** | **0.025** | 0.577 | 0.067 |
| **Anemia** | -0.391 | 0.937 | 0.755 | 0.182 | -0.131 | 0.998 | 0.24 | 0.357 | 0.141 | 0.987 | 0.539 | 0.103 |
| **Elevated AST** | 0.148 | 1 | 0.552 | 0.366 | -0.367 | 0.728 | 0.09 | 0.932 | 0.482 | 0.261 | 0.353 | 0.346 |
| **Elevated ALT** | -0.274 | 0.985 | 0.569 | 0.323 | -0.386 | 0.696 | 0.065 | 0.988 | 0.54 | 0.17 | 0.384 | 0.261 |
| **Hematuria or hemoglobinuria** | -0.045 | 1 | 0.185 | 0.99 | -0.043 | 1 | 0.202 | 0.531 | 0.258 | 0.852 | 0.212 | 0.869 |
| **Evidence of urinary tract infection (microbial growth on urine culture)** | -0.584 | 0.786 | -0.158 | 0.999 | -0.151 | 0.999 | -0.051 | 1 | -0.105 | 1 | 0.101 | 1 |
| **Urinary symptoms** | 0.365 | 0.861 | 0.553 | 0.194 | 0.078 | 1 | 0.248 | 0.079 | 0.431 | 0.125 | 0.37 | 0.168 |

**S1 Table, continued**

|  | **IL-6** |  | **IL-2** |  | **IL-4** |  | **IL-5** |  | **IL-13** |  |
| --- | --- | --- | --- | --- | --- | --- | --- | --- | --- | --- |
|  | **Y - N** | ***P*** | **Y - N** | ***P*** | **Y - N** | ***P*** | **Y - N** | ***P*** | **Y - N** | ***P*** |
| **Age ≥ 50** | 0.271 | 0.786 | 0.115 | 0.912 | -0.5 | 0.953 | 0.079 | 0.968 | 0.347 | 0.803 |
| **Age ≥ 60** | 0.446 | 0.312 | 0.069 | 0.983 | -0.045 | 0.962 | 0.045 | 0.996 | -0.056 | 1 |
| **Gender (male)** | 0.172 | 0.964 | 0.161 | 0.809 | -0.048 | 0.962 | -0.196 | 0.627 | -0.892 | 0.101 |
|  |  |  |  |  |  |  |  |  |  |  |
| **Duration of illness ≤ 4 days** | 0.071 | 1 | **0.394** | **0.048** | -0.045 | 0.965 | 0.041 | 0.997 | -0.25 | 0.923 |
|  |  |  |  |  |  |  |  |  |  |  |
| **Laboratory diagnostics** |  |  |  |  |  |  |  |  |  |  |
| **Positive serology** | 0.243 | 0.954 | -0.209 | 0.557 | 0.073 | 0.846 | -0.08 | 0.964 | -0.03 | 1 |
| **Positive blood smear** | **1.327** | **0.03** | 0.061 | 0.997 | 0.149 | 0.626 | -0.107 | 0.977 | 0.143 | 0.997 |
|  |  |  |  |  |  |  |  |  |  |  |
|  |  |  |  |  |  |  |  |  |  |  |
| **Hospitalized** | 0.546 | 0.258 | 0.243 | 0.455 | -0.064 | 0.902 | 0.163 | 0.734 | 0.75 | 0.201 |
| **Clinical manifestations:** |  |  |  |  |  |  |  |  |  |  |
| **Fever** | 0.604 | 0.401 | 0.324 | 0.695 | 0.152 | 0.78 | 0.099 | 0.995 | 0.9 | 0.518 |
| **Chills** | 0.336 | 0.624 | 0.098 | 0.941 | 0.122 | 0.43 | 0.131 | 0.824 | 0.294 | 0.872 |
| **Fatigue** | -0.641 | 0.063 | -0.258 | 0.325 | -0.104 | 0.561 | 0.039 | 0.998 | 0.378 | 0.73 |
| **Headache** | 0.17 | 0.965 | 0.141 | 0.816 | 0.028 | 0.993 | -0.311 | 0.12 | -0.68 | 0.225 |
| **Myalgias** | -0.11 | 0.996 | 0.027 | 1 | 0.044 | 0.96 | 0.125 | 0.839 | -0.218 | 0.947 |
| **Cough** | 0.47 | 0.406 | 0.23 | 0.527 | -0.0077 | 1 | 0.123 | 0.873 | 0.357 | 0.826 |
| **Nausea** | 0.3 | 0.755 | -0.05 | 0.995 | 0.0038 | 1 | 0.044 | 0.996 | 0.312 | 0.871 |
| **Vomiting** | 0.189 | 0.9861 | 0.013 | 1 | 0.233 | 0.195 | **0.73** | **0.005** | **1.523** | **0.023** |
| **Diarrhea** | -0.348 | 0.816 | -0.08 | 0.996 | -0.027 | 1 | **0.745** | **0.004** | **1.79** | **0.006** |
| **Decreased appetite** | -0.569 | 0.138 | -185 | 0.699 | -0.028 | 0.995 | 0.155 | 0.771 | 0.054 | 1 |
| **Leukopenia** | -0.503 | 0.255 | 0.141 | 0.853 | -0.072 | 0.865 | 0.057 | 0.993 | 0.018 | 1 |
| **Thrombocytopenia** | **0.929** | **0.031** | 0.496 | 0.186 | -0.005 | 1 | 0.001 | 1 | -0.533 | 0.864 |
| **Anemia** | 0.568 | 0.356 | 0.274 | 0.415 | -0.158 | 0.381 | -0.212 | 0.657 | -0.416 | 0.809 |
| **Elevated AST** | -0.199 | 0.977 | 0.019 | 1 | 0.06 | 0.968 | 0.314 | 0.35 | 0.572 | 0.61 |
| **Elevated ALT** | 0.123 | 0.998 | -0.102 | 0.968 | 0.156 | 0.436 | 0.04 | 0.999 | -0.071 | 1 |
| **Hematuria or hemoglobinuria** | 0.229 | 0.967 | 0.073 | 0.988 | -0.062 | 0.94 | -0.118 | 0.946 | 0.069 | 1 |
| **Evidence of urinary tract infection (microbial growth on urine culture)** | -0.416 | 0.903 | -0.405 | 0.382 | 0.067 | 0.879 | 0.048 | 1 | 0.04 | 1 |
| **Urinary symptoms** | 0.407 | 0.397 | 0.112 | 0.904 | 0.006 | 1 | -0.027 | 0.999 | 0.029 | 1 |
