# Supplementary material for: Pro-inflammatory immune responses are associated with clinical signs and symptoms of human anaplasmosis
Source: PLoS One. 2017 Jun 19;12(6):e0179655. doi: 10.1371/journal.pone.0179655 (PMC5476275; doi:10.1371/journal.pone.0179655)
Supplement: S2 Table — Correlation matrix for the log-transformed cytokine concentrations measured in HA patients in the upper, right corner of the matrix and for the controls in the lower, left corner of the matrix. Values are Spearman rank correlations. Cells are color-coded according to the p-value. (DOCX) [file pone.0179655.s002.docx]

**S2 Table.** **Correlations among circulating cytokines in HA and among control subjects.** Correlation matrix for the log-transformed cytokine concentrations measured in HA patients in the upper, right corner of the matrix and for the controls in the lower, left corner of the matrix. Values are Spearman rank correlations. Cells are color-coded according to the p-value.

|  | **IFN-γ** | **IL-10** | **IL-12p70** | **IL-1β** | **IL-8** | **TNF-α** | **IL-6** | **IL-2** | **IL-4** | **IL-5** | **IL13** |  |  | **p-value** |
| --- | --- | --- | --- | --- | --- | --- | --- | --- | --- | --- | --- | --- | --- | --- |
| **IFN-γ** | 1 | 0.120 | 0.708 | 0.452 | 0.438 | 0.230 | 0.485 | 0.690 | 0.0091 | 0.234 | -0.126 |  |  | <0.0001 |
|  |  |  |  |  |  |  |  |  |  |  |  |  |  | <0.001 |
| **IL-10** | 0.229 | 1 | 0.059 | 0.663 | 0.617 | 0.727 | 0.605 | 0.0631 | 0.291 | 0.112 | 0.376 |  |  | <0.01 |
|  |  |  |  |  |  |  |  |  |  |  |  |  |  | ≤0.05 |
| **IL-12p70** | 0.188 | 0.286 | 1 | 0.290 | 0.379 | 0.253 | 0.294 | 0.605 | -0.108 | 0.715 | 0.290 |  |  | >0.05 |
|  |  |  |  |  |  |  |  |  |  |  |  |  |  |  |
|  |  |  |  |  |  |  |  |  |  |  |  |  |  |  |
| **IL-1β** | 0.111 | 0.253 | 0.0146 | 1 | 0.742 | 0.695 | 0.691 | 0.323 | 0.309 | 0.198 | 0.263 |  |  |  |
|  |  |  |  |  |  |  |  |  |  |  |  |  |  |  |
| **IL-8** | 0.121 | 0.156 | 0.0975 | 0.193 | 1 | 0.704 | 0.658 | 0.332 | -0.0425 | 0.303 | 0.210 |  |  |  |
|  |  |  |  |  |  |  |  |  |  |  |  |  |  |  |
| **TNF-α** | 0.315 | 0.425 | 0.0803 | 0.114 | 0.302 | 1 | 0.596 | 0.0997 | 0.140 | 0.109 | 0.331 |  |  |  |
|  |  |  |  |  |  |  |  |  |  |  |  |  |  |  |
| **IL-6** | 0.233 | 0.322 | 0.0249 | 0.165 | 0.256 | 0.398 | 1 | 0.332 | 0.0880 | -0.0834 | -0.0171 |  |  |  |
|  |  |  |  |  |  |  |  |  |  |  |  |  |  |  |
| **IL-2** |  |  |  |  |  |  |  | 1 | -0.074 | 0.353 | -0.0607 |  |  |  |
|  |  |  |  |  |  |  |  |  |  |  |  |  |  |  |
| **IL-4** |  |  |  |  |  |  |  |  | 1 | 0.0262 | 0.193 |  |  |  |
|  |  |  |  |  |  |  |  |  |  |  |  |  |  |  |
| **IL-5** |  |  |  |  |  |  |  |  |  | 1 | 0.521 |  |  |  |
|  |  |  |  |  |  |  |  |  |  |  |  |  |  |  |
| **IL-13** |  |  |  |  |  |  |  |  |  |  | 1 |  |  |  |
|  |  |  |  |  |  |  |  |  |  |  |  |  |  |  |
