# Supplementary material for: Pro-inflammatory immune responses are associated with clinical signs and symptoms of human anaplasmosis
Source: PLoS One. 2017 Jun 19;12(6):e0179655. doi: 10.1371/journal.pone.0179655 (PMC5476275; doi:10.1371/journal.pone.0179655)
Supplement: S3 Table — Summary of the PCA analysis based on concentrations of the Th1/pro-inflammatory cytokines measured in the HA patients (n = 80). Values are the eigenvectors for each cytokine on the retained component scores. Proportions of the variation in the cytokine data explained by the component scores are also indicated. (DOCX) [file pone.0179655.s003.docx]

**S3 Table.** **PCA results for Th1/pro-inflammatory cytokines, HA patients.** Summary of the PCA analysis based on concentrations of the Th1/pro-inflammatory cytokines measured in the HA patients (n=80). Values are the eigenvectors for each cytokine on the retained component scores. Proportions of the variation in the cytokine data explained by the component scores are also indicated.

| Cytokine | Component score 1,  proportion= 0.599 | Component score 2,  proportion = 0.205 |
| --- | --- | --- |
| IFN-γ | 0.2927 | 0.5805 |
| IL-10 | 0.3915 | -0.3571 |
| IL-12p70 | 0.2053 | 0.6887 |
| IL-1β | 0.4335 | -0.1245 |
| IL-8 | 0.4248 | -0.0064 |
| TNF-α | 0.4156 | -0.1998 |
| IL-6 | 0.4217 | -0.0753 |
