# Supplementary material for: Pro-inflammatory immune responses are associated with clinical signs and symptoms of human anaplasmosis
Source: PLoS One. 2017 Jun 19;12(6):e0179655. doi: 10.1371/journal.pone.0179655 (PMC5476275; doi:10.1371/journal.pone.0179655)
Supplement: S4 Table — Summary of the PCA analysis based on concentrations of the Th1/Th2 cytokines measured in the HA patients (n = 41). Values are the eigenvectors for each cytokine on the retained component scores. Proportions of the variation in the cytokine data explained by the component scores are also indicated. (DOCX) [file pone.0179655.s004.docx]

**S4 Table.** **PCA results for Th1/Th2 cytokines, HA patients.** Summary of the PCA analysis based on concentrations of the Th1/Th2 cytokines measured in the HA patients (n=41). Values are the eigenvectors for each cytokine on the retained component scores. Proportions of the variation in the cytokine data explained by the component scores are also indicated.

| Cytokine | Component score 1,  proportion = 0.388 | Component score 2,  proportion = 0.221 | Component score 3,  proportion = 0.175 | Component score 4,  proportion = 0.0855 |
| --- | --- | --- | --- | --- |
| IFN-γ | 0.2423 | 0.2857 | -0.3944 | 0.3119 |
| IL-10 | 0.3991 | -0.2365 | 0.1916 | -0.0202 |
| IL-12p70 | 0.1319 | 0.5826 | -0.0649 | 0.0012 |
| IL-1β | 0.4414 | -0.1172 | 0.0415 | 0.0762 |
| IL-8 | 0.4171 | -0.0083 | -0.0484 | -0.2588 |
| TNF-α | 0.3958 | -0.1561 | 0.1356 | -0.2149 |
| IL-6 | 0.3965 | -0.1894 | -0.2093 | -0.0438 |
| IL-2 | 0.2205 | 0.3537 | -0.3670 | 0.1804 |
| IL-4 | 0.0987 | -0.1423 | 0.3134 | 0.8542 |
| IL-5 | 0.0870 | 0.4760 | 0.4106 | -0.1366 |
| IL-13 | 0.1263 | 0.2774 | 0.5795 | -0.0246 |
