# Supplementary material for: Pro-inflammatory immune responses are associated with clinical signs and symptoms of human anaplasmosis
Source: PLoS One. 2017 Jun 19;12(6):e0179655. doi: 10.1371/journal.pone.0179655 (PMC5476275; doi:10.1371/journal.pone.0179655)
Supplement: S5 Table — Summary of the PCA analysis based on concentrations of the Th1/pro-inflammatory cytokines measured in the controls (n = 1000). Values are the eigenvectors for each cytokine on the retained component scores. Proportions of the variation in the cytokine data explained by the component scores are also indicated. (DOCX) [file pone.0179655.s005.docx]

**S5 Table.** **PCA results for Th1/pro-inflammatory, controls.** Summary of the PCA analysis based on concentrations of the Th1/pro-inflammatory cytokines measured in the controls (n=1000). Values are the eigenvectors for each cytokine on the retained component scores. Proportions of the variation in the cytokine data explained by the component scores are also indicated.

| Cytokine | Component score 1,  proportion = 0.307 | Component score 2, proportion = 0.169 | Component score 3,  proportion = 0.139 | Component score 4,  Proportion = 0.130 |
| --- | --- | --- | --- | --- |
| IFN-γ | 0.4060 | -0.1288 | -0.1790 | -0.3879 |
| IL-10 | 0.5064 | 0.4047 | 0.0066 | -0.1258 |
| IL-12p70 | 0.2819 | 0.7430 | 0.0062 | 0.2226 |
| IL-1β | 0.2124 | -0.1171 | 0.8863 | -0.335 |
| IL-8 | 0.2791 | -0.2463 | 0.2784 | 0.8191 |
| TNF-α | 0.4671 | -0.3069 | -0.2391 | -0.0173 |
| IL-6 | 0.3974 | -0.3149 | -0.2181 | 0.0231 |
