# Supplementary material for: Pro-inflammatory immune responses are associated with clinical signs and symptoms of human anaplasmosis
Source: PLoS One. 2017 Jun 19;12(6):e0179655. doi: 10.1371/journal.pone.0179655 (PMC5476275; doi:10.1371/journal.pone.0179655)
Supplement: S6 Table — Summary of the PCA analysis based on concentrations of the Th1/pro-inflammatory cytokines measured in the HA patients and the controls (n = 1080). Values are the eigenvectors for each cytokine on the retained component scores. Proportions of the variation in the cytokine data explained by the component scores are also indicated. (DOCX) [file pone.0179655.s006.docx]

**S6 Table.** **PCA results for Th1/pro-inflammatory, HA patients and controls.** Summary of the PCA analysis based on concentrations of the Th1/pro-inflammatory cytokines measured in the controls (n=1080). Values are the eigenvectors for each cytokine on the retained component scores. Proportions of the variation in the cytokine data explained by the component scores are also indicated.

| Cytokine | Component score 1,  prop = 0.696 | Component score 2  Prop = 0.095 |
| --- | --- | --- |
| IFN-γ | 0.4064 | 0.2150 |
| IL-10 | 0.4104 | 0.0333 |
| IL-12p70 | 0.3269 | 0.7667 |
| IL-1β | 0.3376 | -0.4963 |
| IL-8 | 0.3911 | -0.0136 |
| TNF-α | 0.3961 | -0.1770 |
| IL-6 | 0.3683 | -0.2952 |
